# Evolution of the Calcium Paradigm: The Relation between Vitamin D, Serum Calcium and Calcium Absorption

**DOI:** 10.3390/nu2090997

**Published:** 2010-09-16

**Authors:** Borje E. Christopher Nordin

**Affiliations:** 1 Endocrine & Metabolic Unit, Royal Adelaide Hospital, Adelaide 5000, South Australia, Australia; Email: christopher.nordin@health.sa.gov.au; 2 Discipline of Medicine, University of Adelaide, Adelaide 5000, South Australia, Australia

**Keywords:** : calcium, vitamin D, parathyroid hormone, osteoporosis, osteomalacia

## Abstract

Osteoporosis is the index disease for calcium deficiency, just as rickets/osteomalacia is the index disease for vitamin D deficiency, but there is considerable overlap between them. The common explanation for this overlap is that hypovitaminosis D causes malabsorption of calcium which then causes secondary hyperparathyroidism and is effectively the same thing as calcium deficiency. This paradigm is incorrect. Hypovitaminosis D causes secondary hyperparathyroidism at serum calcidiol levels lower than 60 nmol/L long before it causes malabsorption of calcium because serum calcitriol (which controls calcium absorption) is maintained until serum calcidiol falls below 20 nmol/L. This secondary hyperparathyroidism, probably due to loss of a “calcaemic” action of vitamin D on bone first described in 1957, destroys bone and explains why vitamin D insufficiency is a risk factor for osteoporosis. Vitamin D thus plays a central role in the maintenance of the serum (ionised) calcium, which is more important to the organism than the preservation of the skeleton. Bone is sacrificed when absorbed dietary calcium does not match excretion through the skin, kidneys and bowel which is why calcium deficiency causes osteoporosis in experimental animals and, by implication, in humans.

## 1. Instruction

According to current knowledge, cholecalciferol (vitamin D_3_) is synthesised in the skin from 7-dehydrocholesterol under the influence of sunlight, conveyed to the liver to be converted into 25-hydroxy cholecalciferol (calcidiol) which is conveyed to be kidneys for further conversion into 1,25-dihydroxy-cholecalciferol (calcitriol) or downgraded to 24,25-dihydroxycholecalciferol. Calcidiol circulates in plasma in nanomolar concentrations, mostly bound to D-binding protein (DBP), and calcitriol in picommolar concentrations, also largely bound to DBP. However, because the vitamin D receptor has a much higher affinity for calcitriol than for calcidiol, the former has much the more powerful action on calcium absorption and bone resorption than the latter and is frequently referred to as the “active metabolite” of vitamin D or the “hormonal form”. It should be noted that the hypercalcaemia of vitamin D over-dosage is probably more due to high bone resorption than to high calcium absorption.

The index disease for vitamin D deficiency is rickets in children and osteomalacia in adults, just as the index disease for calcium deficiency is osteoporosis [1]. But there is some overlap between these two deficiency-states. The explanation for both the difference and the overlap between them is not well understood by many workers in the field who appear to be operating in an outdated paradigm. This is best understood by looking at the history of the subject which was reviewed by Nordin in 1960 [2] but now tends to be overlooked. 

Briefly, the disease of rickets/osteomalacia, first described in the 17th century, became rampant in industrialised countries during the 19th century but was not clearly distinguished from osteoporosis until 1885 [3] when Pommer showed that it was not due to removal of calcium from bone but failure to deposit calcium in new bone. It was already known to be associated with lack of sunlight and to be curable with fish oils but was generally also thought to be a form of calcium deficiency until the distinction between calcium deficiency, causing osteoporosis, and sunlight deficiency, causing rickets, was clearly established in the early twentieth century by German researchers [4]. The active principle in fish oils was later identified as vitamin D by Mellanby in 1919 [5]. For a brief period, the distinction between calcium deficiency and vitamin D deficiency was the accepted paradigm but when it was shown in the 1920’s that rickets was associated with malabsorption of calcium [6], it came to be thought that malabsorption of calcium was the cause of the disease and the pathogenesis of osteoporosis was returned to limbo. At this point, Fuller Albright introduced the idea that rickets/osteomalacia could be due to deficiency of vitamin D or calcium and declared that osteoporosis was due to a failure of osteoblasts to lay down new bone [7]. He brilliantly predicted and explained the adverse effect on bone formation of what he called the S-hormone (Sugar-hormone), which later proved to be hydrocortisone, but he was wrong in extending this concept to postmenopausal bone loss which is due to an increase in bone resorption. In fact, when bone formation rate became measurable with radiocalcium it was shown to be no lower in osteoporotic than normal women but such was his influence that his paradigm still carries considerable weight. Yet a seminal Swedish paper in 1955 [8] showed that vitamin D did more than simply regulate calcium absorption—it also had an independent calcaemic action which helped to maintain plasma calcium in a manner that could not be attributed to its action on calcium absorption; this work explained the difference between calcium deficiency and vitamin D deficiency shown 50 years earlier in Germany. It is now clear that negative calcium balance, whether due to low intake, low absorption or high excretion of calcium, causes osteoporosis because the maintenance of the plasma calcium concentration is more important to the organism, for neuromuscular and other reasons, than is the preservation of the skeleton; the calcium deficit is made up by breaking down bone rather than by lowering the calcium concentration in the tissue fluids. The role of parathyroid hormone in this process is still the subject of controversy but the sequence of events from hypovitaminosis D to rickets/osteomalacia as already understood in the 1960’s remains valid today. Because of the loss of its calcaemic action (the precise mechanism of which is not fully understood), lack of vitamin D causes a fall in the calcium level in the tissue fluids, secondary hyperparathyroidism and hypophosphataemia, to which malabsorption of phosphate also contributes. This hypophosphataemia lowers the tissue fluid Ca × P product which delays the mineralisation of new bone and calcification of growth cartilage which are the essential features of osteomalacia and rickets. 

The reason for the overlap between the two conditions is also now apparent in that the secondary hyperparathyroidism which appears early in vitamin D insufficiency increases bone resorption and so accelerates the osteoporotic process but at the same time maintains the serum calcitriol (and therefore calcium absorption) until the D-deficiency is so severe that the calcitriol level falls from lack of substrate which leads to the malabsorption of calcium and phosphate which are a feature of rickets/osteomalacia [9]. Every degree of vitamin D insufficiency can be seen in the histology [10] and serum biochemistry [11,12] of hip fracture patients who generally suffer from malabsorption of calcium [13] due to varying combinations of low serum calcitriol and age-related “resistance” to calcitriol (see below). Conversely, in young animals with a high calcium requirement for growth, it is possible for severe calcium deficiency to cause hypocalcaemia and consequent rickets/osteomalacia even when vitamin D status is normal [14]. Thus the overlap between calcium and vitamin D deficiency can be explained in both directions. 

This “new” paradigm, first described in 1960 [2], is heavily dependent on a knowledge of the internal relationships between serum PTH, calcidiol, calcitriol and calcium absorption, all of which can be measured. The only one of these measurements that is at all controversial is that of calcium absorption which used to be determined by balance procedures which are now considered too expensive and time-consuming even for research purposes and quite out of the question for routine use. Calcium balance studies have therefore been largely superseded by a simple radiocalcium procedure pioneered by Avioli [15] but revised, developed and applied over the last forty years by Nordin and colleagues, first in Leeds, England and then in Adelaide, Australia. The theoretical basis of this procedure was first set out in 1969 [16] and based on the administration of radiolabelled calcium in 20 mg of calcium carrier to the fasting subject, collection of 6 blood samples over the next 2 hours and calculation of the rates of calcium input and output by deconvolution analysis. The radiocalcium absorption calculated in this way was shown to correlate very significantly with the rate of dietary calcium absorption (corrected for intake) in simultaneous calcium balances [17] and very highly with calcium absorption calculated by the double isotope method [18]. The results were shown to fall with age [19], to be high in hyperparathyroidism and calcium-stone disease and low in osteomalacia and renal failure [17], and (as said above) low in women with hip fractures [13]. Radiocalcium absorption was later shown to rise on treatment of osteoporosis [20] and renal failure [21] with 1α-hydroxyvitamin D and to be related to the serum calcitriol level [22,23]. 

Further experience showed that the plasma radioactivity 60 minutes after the tracer dose (the peak value) was so highly correlated with the absorption rate calculated from the 6 blood samples (*r* values around 0.9) [18], that this single blood sample was adopted as a valid basis for calculating calcium absorption as an hourly fractional rate from a conversion equation [24] which was later further refined to correct for body weight [25]. This simplified test has since been shown to behave in the same way as the full procedure; it correlates in the same way with the serum calcitriol and responds in the same way to calcitriol therapy [26,27,28]. It has demonstrated that the fall in calcium absorption with age cannot be accounted for by a fall in serum calcitriol [29] as has also been reported in aged rats [30]. A longitudinal study across the menopause also showed a fall in calcium absorption at menopause independent of serum calcitriol [31] as had previously been shown in ovariectomised rats [32]. The practical clinical significance of this test is shown by the fact that calcium supplementation is much more effective in suppressing bone turnover markers in women with normal radiocalcium absorption than in those with low radiocalcium absorption [33].

It needs to be emphasised that the small amount of calcium carrier used in this absorption test is crucial to its validity. The low carrier ensures that absorption occurs quickly and over a limited length of small intestine which yields the clean curve that would be expected from absorption at a single point and which is analysable on that assumption. The more carrier that is used, the lower the fraction absorbed, the longer the length of small intestine involved and the less analysable the subsequent curve becomes since it is in fact a family of curves. The use of 250 mg or more of calcium carrier is superficially attractive because that represents a normal meal but it cannot be done with a single isotope—it requires a double isotope procedure. Moreover, the high carrier version of the single isotope test [34] is not only insensitive but must carry a large error and has never been shown to correlate with serum calcitriol or validated in the same way as the low carrier test. What an absorption test should provide is reliable information about the active transport component of calcium absorption. The assumption, borne out by combined isotope and balance studies [25], is that it reflects the “normality” of calcium absorption at all intakes. Net absorbed calcium can be corrected for intake by calculation from calcium balance studies on normal subjects and is largely mediated by a saturable transport process [17] and correlated with radiocalcium absorption. Thus a radiocalcium absorption rate of 0.75 per hour—the normal mean [31]—signifies that absorption is probably normal for intake at all physiological intakes. The test is not a measure of serum calcitriol as such but of calcium absorptive status. The main determinant of calcium absorption is of course the serum calcitriol level but ageing and menopause are both associated with a “resistance” to calcitriol and this is particularly true in osteoporotic women with vertebral fractures [35]. Despite this, the calcium malabsorption in such patients can be corrected by calcitriol administration and there is a dose-response relationship between the dose of calcitriol and the radiocalcium absorption level which is established within a few days [36] whereas Vitamin D alone has little or no effect on calcium absorption in women with vertebral fractures [37]. It may be possible to show a weak positive univariate correlation between calcium absorption and serum calcidiol due to the internal correlation between serum calcidiol and calcitriol, generally about 0.3 (P < 0.001), which is simply because the former is the substrate for the latter; when serum calcitriol is included in the equation, serum calcidiol invariably drops out. It is most unfortunate that this spurious correlation between calcidiol and calcium absorption [38] has been accepted so easily by some sections of the scientific community when it is not only incorrect but offers a misleading explanation of the secondary hyperparathyroidism which is such an important feature of hypovitaminosis D.

As far as vitamin D requirement is concerned, with a half-life of about 20 days, the serum calcidiol will sooner or later reach an equilibrium value on any particular dose of vitamin D sooner or later, as elegantly shown by Stamp many years ago [39]. The rise depends largely on the starting value. The vitamin D requirement must be the amount required to achieve a desirable equilibrium value. We regard the intervention value as 60 nmol/L (because that is the value below which bone turnover markers to rise [40]) and the desirable level as 80–100 nmol/L. The latter can be achieved in a matter of weeks in most subjects on a dose of 1,000 units daily of cholelcalciferol but we are aware of the view that the optimal level should perhaps be higher, and it is possible that 2,000 units a day will ultimately come to be regarded as the correct standard dose, but we see no merit in large loading doses since vitamin D-insufficiency can hardly be regarded as an emergency. We regard 25OHD values between 15 and 60 nmol/L as vitamin D “insufficiency” and values below 15 nmol/L as true “deficiency” which ultimately leads to rickets/osteomalacia [9]. However, we are satisfied from the evidence that prevention of bone loss and fractures in the elderly requires a combination of calcium with vitamin D [41,42,43] and we now believe that the current calcium allowance for postmenopausal women of 1,300 mg [44] should probably be 1,500 mg [45]. However, it has to be said that the use of calcium balances to calculate calcium requirement is complicated by uncertainty about dermal losses. The FAO/WHO Requirements and Allowances [44] assumed a skin loss of 60 mg daily, as reported by Charles *et al.* [46], but Charles has now reported a technical error in his procedure and believes the correct figure should be about 30–40 mg [personal communication]. It is therefore important to seek further evidence on dermal losses of calcium. On the other hand, Nordin’s review of 32 calcium trials in postmenopausal women [47] provides empirical evidence that their average requirement is about 1,300 mg which suggests that the allowance should in fact be about 1,500 mg.

## 2. Conclusions

It follows from this brief review that the “new” paradigm introduced by Nordin in 1960 [2] fits the available facts better than the “old” paradigm which fails to explain the differences and similarities between calcium and vitamin D deficiency. Importantly, the widespread but misleading concept that malabsorption of calcium *per se* causes secondary hyperparathyroidism needs to be corrected [28]. This confusion of paradigms in the scientific community has allowed the pharmaceutical industry to penetrate the market with expensive remedies for the treatment of osteoporosis when the correct policy should surely be bone densitometry of all women at menopause and the adoption of appropriate lifestyle measures by those with “osteopenia” [48] to prevent them developing osteoporosis.
